# Analysis of Pan-Cancer Revealed the Immunological and Prognostic Potential of CBX3 in Human Tumors

**DOI:** 10.3389/fmed.2022.869994

**Published:** 2022-04-28

**Authors:** Haitao Xu, Caihong Jiang, Dangui Chen, Youzhi Wu, Jia Lu, Long Zhong, Fusheng Yao

**Affiliations:** ^1^Department of Hematology, Anqing Municipal Hospital, Anqing Hospital Affiliated to Anhui Medical University, Anqing, China; ^2^Department of Pediatric Surgery, Anqing Municipal Hospital, Anqing Hospital Affiliated to Anhui Medical University, Anqing, China

**Keywords:** CBX3, pan-cancer, prognosis, immune cell infiltration, TMB, MSI

## Abstract

Chromobox protein homolog 3 (CBX3) has been recognized as a member of the heterochromatin protein 1 family and participate in transcriptional activation or inhibition, cell differentiation and growth. Despite more and more evidence shows that CBX3 has a critical function in the development of some tumors, no systematic extensive analysis of CBX3 has been reported. Thus, we intended to examine the prognostic significance of CBX3 in 33 tumors and investigate its potential immune function. We employed several bioinformatics methods to explore the potential carcinogenic impact of CBX3 premised on the data sets collected from tumor genome maps, human protein maps, cBioPortal, and genotype tissue expression. The approaches include assessing the link between CBX3 and prognosis of different tumors, immune cell infiltration, micro-satellite instability (MSI), DNA methylation, and tumor mutational burden (TMB). The outcomes illustrated that CBX3 was increasingly expressed in 29 tumors. Moreover, CBX3 exhibited a negative correlation with the prognosis of many tumors. The expression of CBX3 was linked to MSI in 12 tumors and TMB in 16 tumors. In 24 tumors, the expression of CBX3 was linked to DNA methylation. Moreover, the CBX3 expression exhibited a negative relationship with the infiltration level of the majority of immune cells, but showed a positive link to T gamma delta cells, central memory T cells, and T helper cells, especially when invading breast carcinoma, thymic carcinoma, colon carcinoma, cutaneous melanoma, endometrial carcinoma, and lung squamous carcinoma. Our research indicates that CBX3 might be used as a prognostic indicator for different malignant tumors due to its function in tumor genesis as well as tumor immunity.

## Introduction

Tumors are the leading cause of death, which threaten human health and impose a huge financial liability on society (1). Regrettably, the incidence of new tumor case diagnoses is still rising steadily, and the harm caused by it is intensifying. Currently, the treatment strategies of cancer mainly comprise immunotherapy, targeted therapy, radiotherapy, chemotherapy, and surgery (1). While these therapeutic interventions take effect in the clinical treatment of tumors, to a certain extent, the prognosis and survival rate of patients is still disappointing owing to numerous cases of medication resistance and adverse side effects (2). Thus, there is a pressing need to actively investigate other treatment targets as well as new sensitive tumor biomarkers to diagnose and facilitate the treatment of tumors (3).

Chromobox protein homolog 3(CBX3), which is a well-known member of the heteroprotein 1 (HP1) family recognizes and binds to the histone H3 lysine 9 (H3K9) to recruit a number of functional cofactors (4). Its cell function includes DNA damage repair (5), cell differentiation (6), telomere function (7), and transcriptional regulation (8). Recent research reports have identified the expression and role of CBX3 in different types of human tumors. In prostate cancer, the increased expression of CBX3 may be an independent factor in predicting biochemical recurrence after radical prostatectomy (9). CBX3 can inhibit the transcription of negative cell cycle regulators CDK6 and P21 and promote the proliferation of colorectal cancer cells (10). CBX3 promotes cancer cell proliferation by inhibiting the FBP1 gene in pancreatic cancer cells. Disruption of the CBX3-FBP1 signaling axis would effectively treat pancreatic cancer and prevent aerobic glycolysis (11). Another study showed that CBX3 upregulation suppressed ARHGAP24 expression and increased the amount of active Rac1 in lung adenocarcinoma (LUAD) cells, thereby promoting LUAD progression (12). In addition, other studies have shown that the overexpression of CBX3 in osteosarcoma (13), tongue squamous carcinoma (14) and breast cancer (15) can adversely affect the prognosis by promoting the occurrence as well as the development of tumors. However, no clinical trials or related treatments targeting CBX3 have been reported to date.

Still, nowadays, most studies on the function of CBX3 in tumors have focused mainly on specified types of tumors. None of the existing studies has focused on the relationship between CBX3 and pan-cancer. Thus, multiple databases were utilized in this research to examine the expression level of CBX3 in patients with various types of tumors and its relationship with prognosis. These datasets include the TCGA, Human Protein Atlas (HPA), cBioPortal, and Genotype Tissue-Expression (GTEx). Additionally, we assessed the prospective connections between the expression of CBX3 and immune invasion levels, DNA methylation, tumor mutational burden (TMB), and microsatellite instability (MSI) in 33 tumors. In addition, the correlation between CBX3 and immune-related genes and the enrichment of pathways were analyzed to examine the biological roles of CBX3 in tumors. The findings from this research indicate that CBX3 could serve as a prognostic marker for different tumors, and has a crucial function in tumor immunity by influencing tumor-infiltrating immune cells (TIICs), MSI and TMB. This research provides an in-depth understanding of the function of CBX3 in tumor immunotherapy.

## Methods

### Data Processing and Differential Expression Analysis

We searched the TCGA (https://portal.gdc.cancer.gov/) and GTEx (Genotype tissue expression) database from UCSC XENA (https://xenabrowser.net/datapages/) uniformly processed by the Toil. The RNAseq data of the CBX3 gene in TPM format in tumor tissues and matching non-tumor normal tissues were downloaded from the database, and then log2 transformation was performed. Later, R (version 3.6.3) software was utilized to contrast the expression of tumor tissues samples, non-tumor normal tissue samples and paired samples. *P* < 0.05 indicated differential expression between tumor and non-tumor normal tissue. The R package “ggpubr” is utilized to plot boxplots.

To evaluate the effectiveness of CBX3 expression in distinguishing tumors from normal samples, ROC analysis was performed on paired samples using pROC packages. The calculated AUC range from 0.5 to 0.1, indicating an identification potential of 50 to 100%.

### Immunohistochemical (IHC) Staining

To assess the differences of CBX3 in protein expression level, the IHC image was downloaded and analyzed from HPA (http://www.proteinatlas.org/). The image shows the CBX3 protein expression in non-tumor normal tissue and 6 tumor tissue, that include breast cancer (BRCA), colon adenocarcinoma (COAD), stomach adenocarcinoma (STAD), pancreatic adenocarcinoma (PAAD), lung adenocarcinoma (LUAD), and liver hepatocellular carcinoma (LIHC).

### Relationship Among CBX3 Expression and Prognosis and Clinical Phenotype

The clinical phenotypic and survival data were collected from each sample accessed from the TCGA. Three indicators including progression-free interval (PFI), disease-specific survival (DSS), and overall survival (OS) were chosen to assess the link between the expression of CBX3 and the prognosis of patients. The log-rank test and Kaplan-Meier (KM) technique were utilized for survival analysis of each tumor type (*P* < 0.05). The survival curve was charted by the R package “Survival” and “SurvMiner”. In addition, Cox analysis was executed by R package “survival” and “forestplot” to ascertain the Pan-cancer connection between the expression of CBX3 and survival.

Two kinds of clinical phenotypes, i.e., age of patient and stage of tumor were chosen and their link to CBX3 expression was discussed. The participants were categorized into two cohorts, with those aged 60 years designated as the critical value. R packages “LIMMA” and “GGPUBR” were used for correlation analysis of clinical phenotypes. *P* < 0.05 was judged to have a significant difference.

### Correlation of CBX3 Expression With TMB and Tumor MSI

TMB has been recognized as a quantifiable immune response biomarker that signifies the number of mutations in the tumor cells (16). MSI is caused by a DNA mismatch repair defect (MMR) and is linked to patient prognosis (17). The TMB score is computed by a Perl script and adjusted premised on the sum of the length of the exons apart. The MSI grades of all samples were determined according to the somatic mutation data downloaded from TCG, and Spearman rank correlation coefficient was utilized to assess the correlation among CBX3 expression, TMB and MSI. The result is displayed as a radar diagram, generated with R packages “ggradar” and “ggplot2”.

### Relationship Between CBX3 Expression of and Immunity

Single sample gene set enrichment analysis (ssGSEA) is utilized in the computation of the enrichment score of the corresponding gene set for each sample with a given gene set (18). SsGSEA algorithm in immune infiltration computes the enrichment score of 24 kinds of immune cells in each tumor sample with the “GSVA” package in R software. R packages “GGploT2”, “GGpubr”, and “ggExtra” were utilized to assess the link between the levels of CBX3 and the infiltration levels of each immune cell in the tumor (*P* < 0.05 was judged as having a significant difference).

XCELL is a tool utilized for computing the enrichment score of each cell type premised on the gene expression profile (19). The xCELL algorithm was used to compute the stromal score, immune score, and microenvironment score for each of the tumor specimens. The correlation between CBX3 levels and the above scores of each tumor sample was assessed using the R package “Immunedeconv” and the “GGploT2” package for the visualization of the results.

In addition, we utilized the R package “Limma” to evaluate the correlation between CBX3 and immune-related genes, including immune checkpoint, immune activation, immune suppression gene, chemokines and chemokine receptor proteins. The “Reshape2” and “RColorBreyer” packages are respectivly utilized for visualizing the outcomes.

### Correlation Between CBX3 Expression and DNA Methylation

DNA methylation is a well-recognized kind of DNA chemical alteration. Premised on its vital function in regulating gene transcription, DNA methylation might be carcinogenic (20). The link between the expression of CBX3 and the DNA methylation of each of the tumors was evaluated utilizing the HM450 methylation data from cBioPortal (www.cbioportal.org). KM survival analysis was utilized to assess CBX3 methylation and OS. *P* < 0.05 was judged as having a significant difference.

### Biological Significance of CBX3 Expression in Tumors

The gene set enrichment analysis (GSEA) was carried out to study the biological role of CBX3 in tumors. We then downloaded the gene ontology (GO) and Kyoto encyclopedia gene and genome (KEGG) genetic set from GSEA official webpage (https://www.gsea-msigdb.org/gsea/downloads.jsp). Enable R packages “Limma”, “org.Hs.eg.db”, “clusterProfiler”, and “enrichPlot” to be utilized for functional analysis.

### Statistical Analysis

Normalization of all gene expression data was done utilizing LOG2 transformation. The cancer tissues and non-tumor normal tissues were contrasted by two groups of *T*-test; *P* < 0.05 indicates results that are statistically significant. The log-rank test, KM curves, as well as, Cox proportional risk regression models were utilized for all the survival analyses performed in this research. Spearman's test was utilized to examine the link between the two variables. *P* < 0.05 was judged as having a significant difference. All the statistical analyses conducted in this research were processed by R software (version 3.6.3).

## Results

### Expression of CBX3 Between Tumor and Non-tumor Normal Tissue Specimens

**Figure 1:**
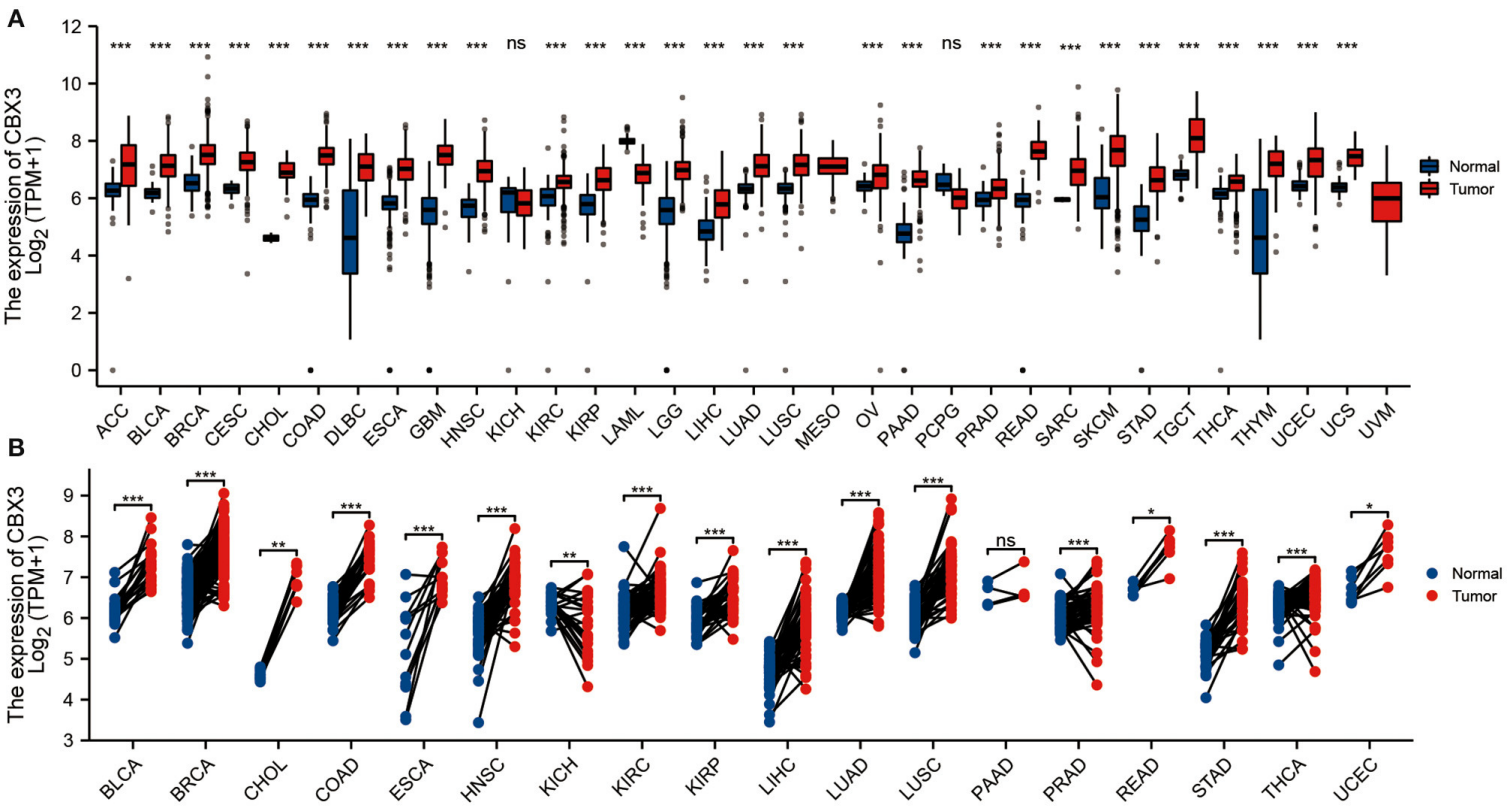
Expression of CBX3. **(A)** Contrast of the expression of CBX3 between tumor samples and non-tumor normal specimens. **(B)** Contrast of CBX3 expression between tumor specimens and paired non-tumor normal specimens. *P < 0.05, **P < 0.01, ***P < 0.001.

To evaluate the CBX3 expression in pan-cancer, we utilized R software to jointly assess the RNA sequencing data in both the GTEx and TCGA. The differential expression patterns of CBX3 in 33 tumors and non-tumor normal tissues are illustrated in Figure 1A. In addition to those tumors without normal tissue data to use, considerable differences in CBX3 expression were identified among 29 tumor tissues and non-tumor normal tissues. The expression of CBX3 was increased in 28 tumor tissues but merely decreased in acute myeloid leukemia (LAML). Premised on the TCGA data, we also contrasted the expression level of CBX3 among tumor tissues from 18 types of tumors and the matched non-tumor normal samples (Figure 1B). Among them, we find high expression in 16 kinds of tumors, including endometrial carcinoma of the uterus (UCEC), thyroid cancer (THCA), stomach adenocarcinoma (STAD), rectal adenocarcinoma (READ), prostate adenocarcinoma (PRAD), lung squamous cell carcinoma (LUSC), lung adenocarcinoma (LUAD), liver hepatocellular carcinoma (LIHC), kidney renal clear cell carcinoma (KIRP), kidney renal clear cell carcinoma (KIRC), head and neck squamous cell carcinoma (HNSC), esophageal carcinoma (ESCA), colon adenocarcinoma (COAD), cholangiocarcinoma (CHOL), invasive breast carcinoma (BRCA), and Bladder Urothelial Carcinoma(BLCA).

**Figure 2:**
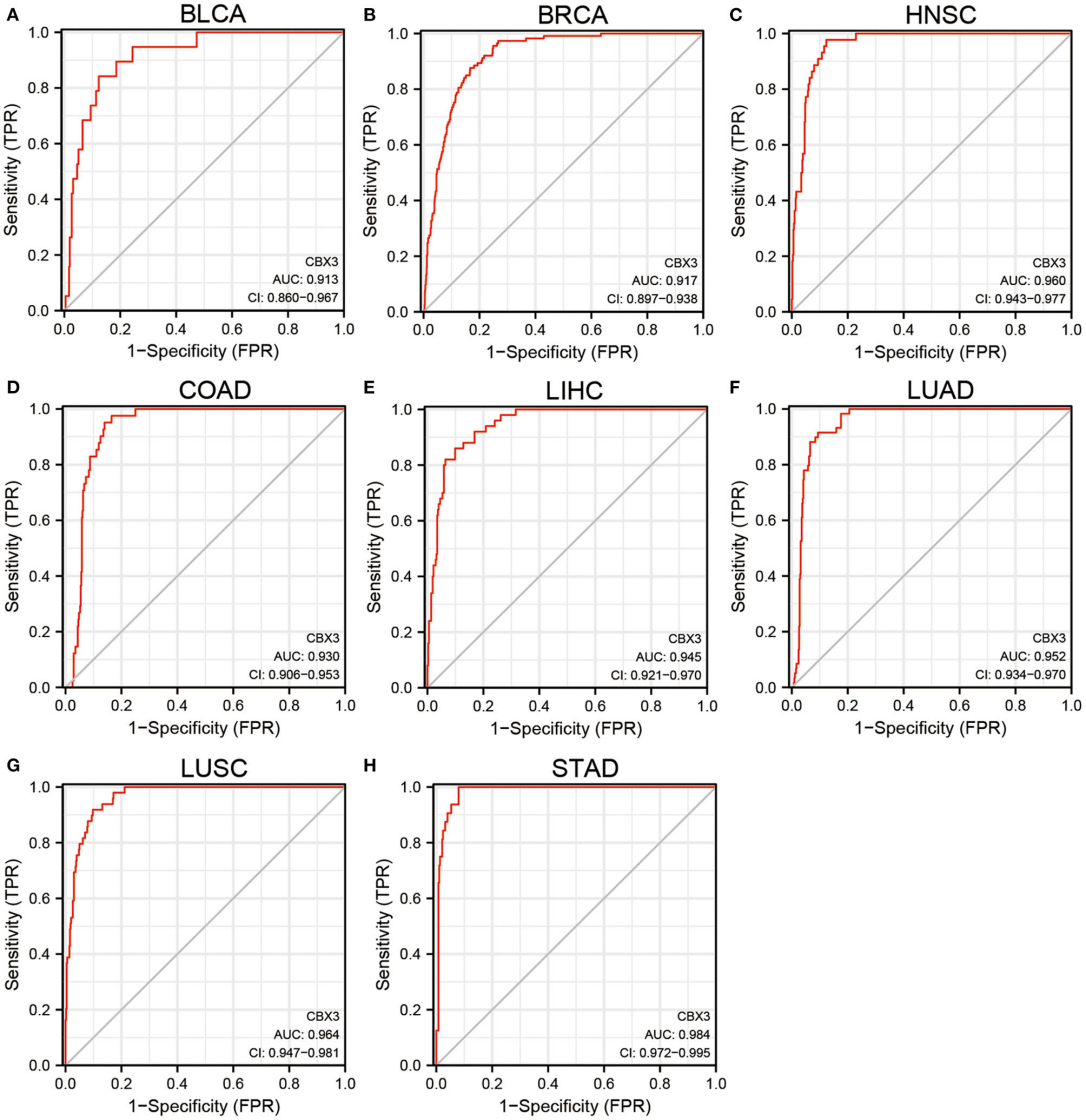
**(A–H)** Comparison of CBX3 expression between tumor and paired non-tumor normal samples. ROC analysis of CBX3 illustrated auspicious capability to differentiate tumors from non-tumor normal tissues.

In addition, CBX3 expression showed a promising ability to distinguish tumor tissue from normal tissue, with AUC values > 0.9 in eight tumors (Figures 2A–H), including BLCA, BRCA, HNSC, COAD, LIHC, LUAD, LUSC, and STAD. In other tumors, except UCEC, the AUC value was > 0.7 (Supplementary Materials are shown in Figure 1).

**Figure 3:**
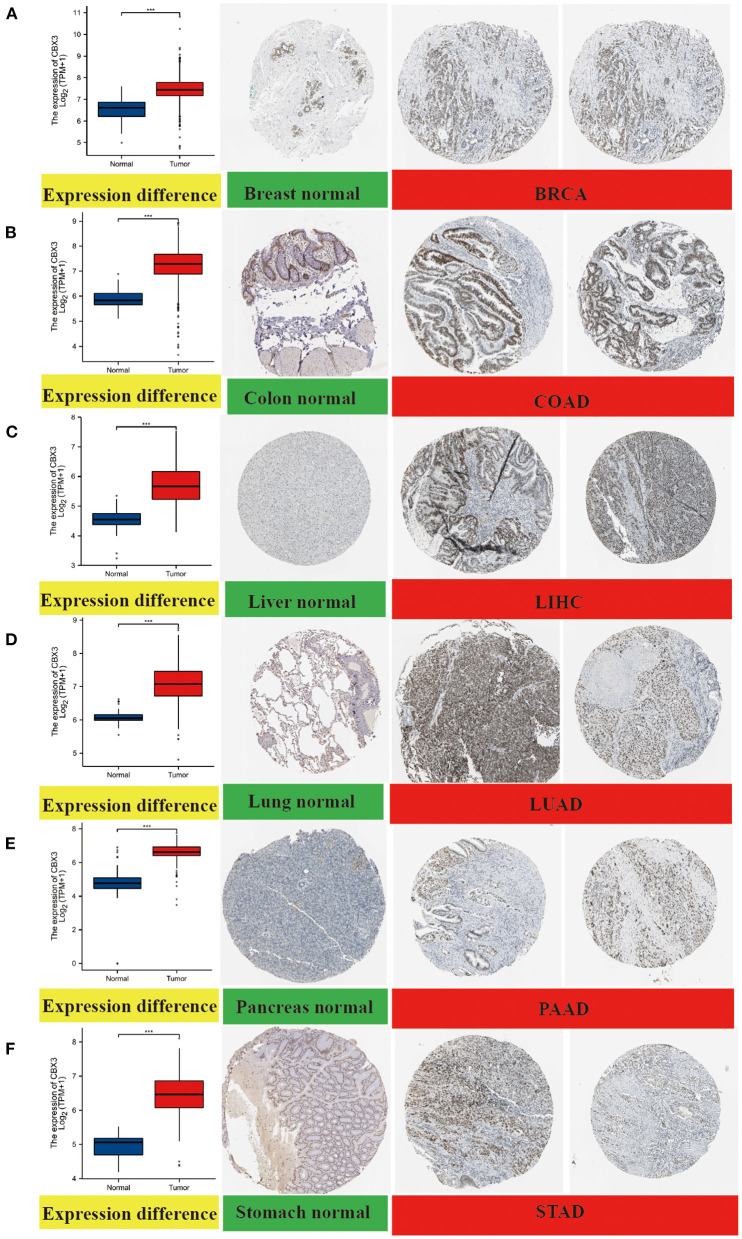
Comparison of CBX3 gene expression between non-tumor normal tissues and tumor tissues (left) and immunohistochemical images of normal tissues (middle) and tumor tissues (right). The expression of CBX3 protein in BRCA, COAD, LIHC, LUA), PAAD and STAD was considerably elevated as opposed to that in non-tumor normal tissues. **(A)** breast, **(B)** colon, **(C)** liver, **(D)** lung, **(E)** pancreas, **(F)** stomach. ***P < 0.001.

In addition, to assess the expression of CBX3 at the protein level, we evaluated the IHC outcomes from the HPA dataset and contrasted these outcomes with the CBX3 gene expression data from the TCGA. As illustrated in Figures 3A–F, the data analysis findings of the two databases are consistent. Normal breast, colon, lung and stomach tissues showed moderate CBX3 IHC staining, whereas tumor tissues depicted strong staining. Additionally, the normal liver and pancreas tissue specimen depicted faint CBX3 staining, whereas tumor tissue depicted sturdy staining.

### The Prognostic Value of CBX3 in Tumors

**Figure 4:**
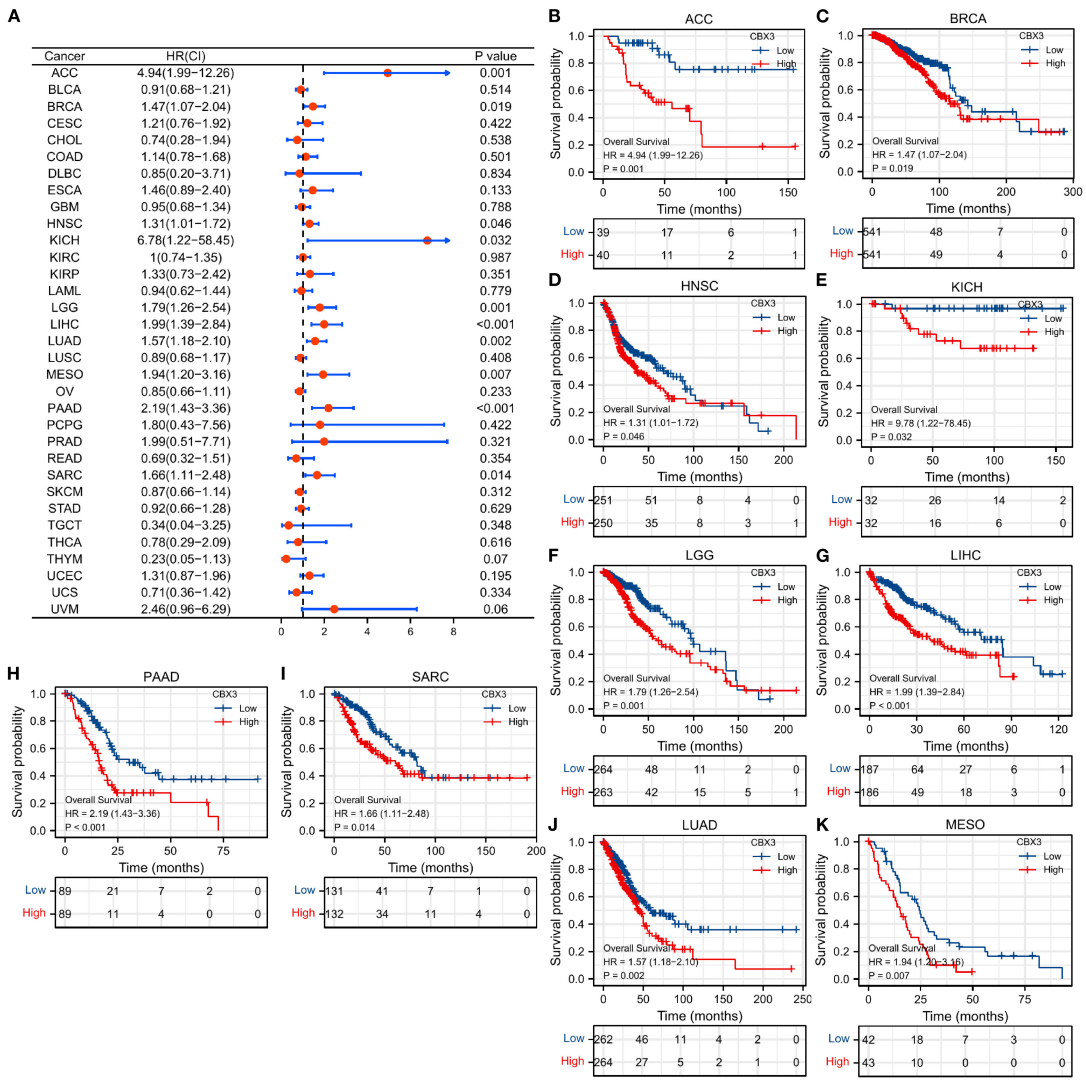
The relationship between the expression of CBX3 and OS. **(A)** Forest map of the link between CBX3 expression and OS in 33 kinds of tumors. **(B–K)** KM analysis of the correlation between CBX3 expression and OS.

To investigate the link between the level of CBX3 expression and prognosis, we conducted a survival association analysis that include PFI, DSS, and OS for each tumor. The Cox proportional risk model analysis illustrated (Figure 4A) CBX3 as a high-risk gene in adrenal cortical carcinoma (ACC), BRCA, HNSC, renal chromophobe cell carcinoma (KICH), low-grade glioma (LGG), LIHC, PAAD, and SARC. KM survival analysis also showed (Figures 4B–K) that patients who had elevated CBX3 levels in these tumors exhibited shorter survival times.

In addition, DSS data analysis (Supplementary Figure 2A) illustrated an undesirable link between high CBX3 expression and prognosis in patients with HNSC, MESO, LUAD, LIHC, KIRP, ACC, LGG, PAAD, and SARC. Also, KM survival analysis showed (Supplementary Figures 2B–J) that patients who had elevated CBX3 levels in these tumors exhibited shorter DSS.

As for the correlation between the expression of CBX3 and PFI, the forest map illustrated that the elevated expression of CBX3 was associated with low-level PFI of HNSC, LGG, LIHC, LUAD, MESO, ACC, and PAAD (Supplementary Figure 3A). KM analysis showed (Supplementary Figures 3B–H) that tumors patients with high expression of CBX3 had poor PFI.

### Correlation Between Expression of CBX3 and Clinical Phenotype of Various Tumors

**Figure 5:**
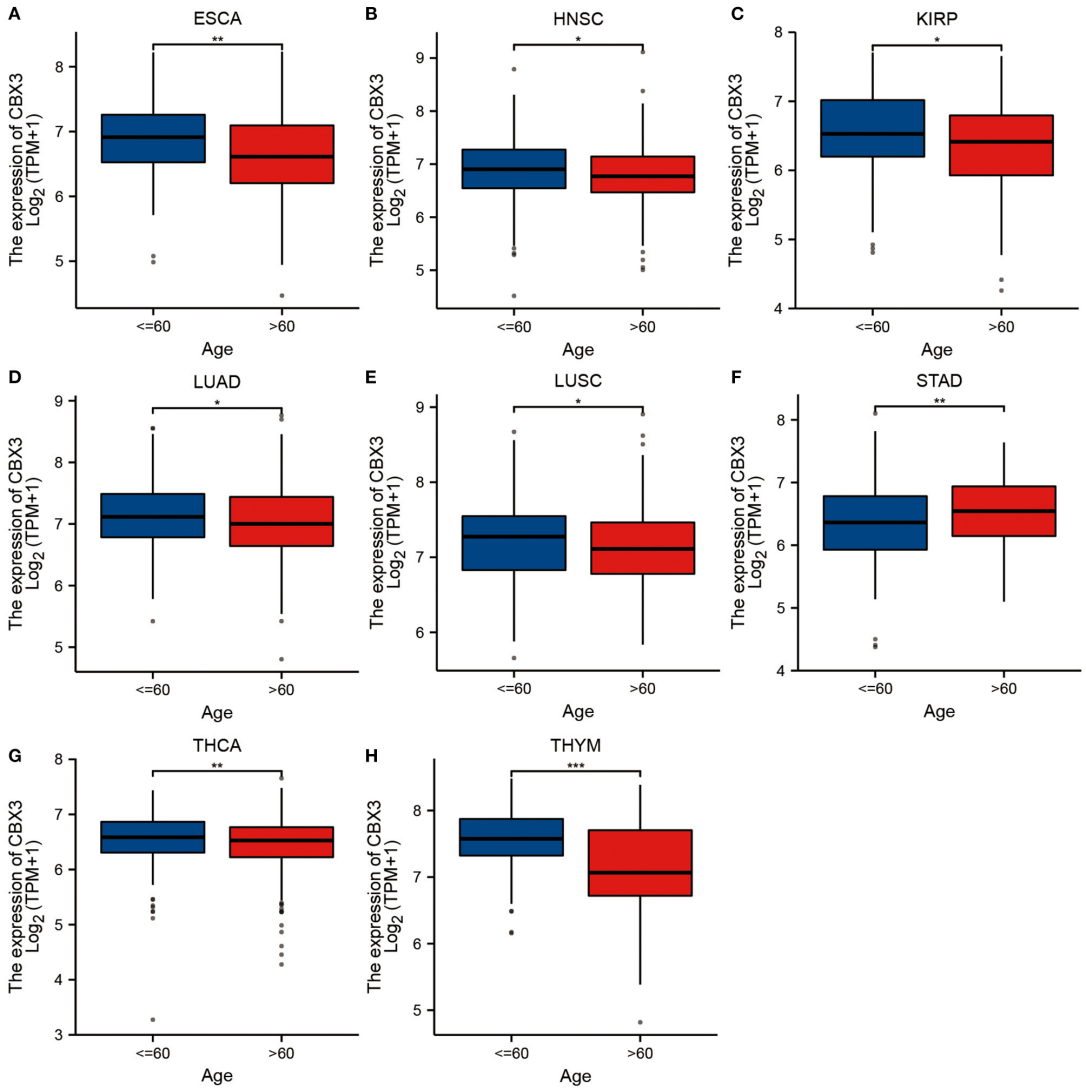
Correlation of CBX3 expression with age in **(A)** ESCA, **(B)** HNSC, **(C)** renal papillary cell carcinoma(KIRP), **(D)** LUAD, **(E)** LUSC, **(F)** STAD, **(G)** THCA and **(H)** thyma.

Next, we examined the differential expression of CBX3 based on the age of patients with each tumor type. Higher CBX3 expression was observed in ESCA (Figure 5A), HNSC (Figure 5B), KIRP (Figure 5C), LUAD (Figure 5D), LUSC (Figure 5E), THCA (Figure 5G), and THYM (Figure 5H) patients aged ≤ 60 years old. Patients with STAD > 60 years of age had a higher CBX3 expression level (Figure 5F). No significant correlation was detected between age and CBX3 expression in other cancer patients.

**Figure 6:**
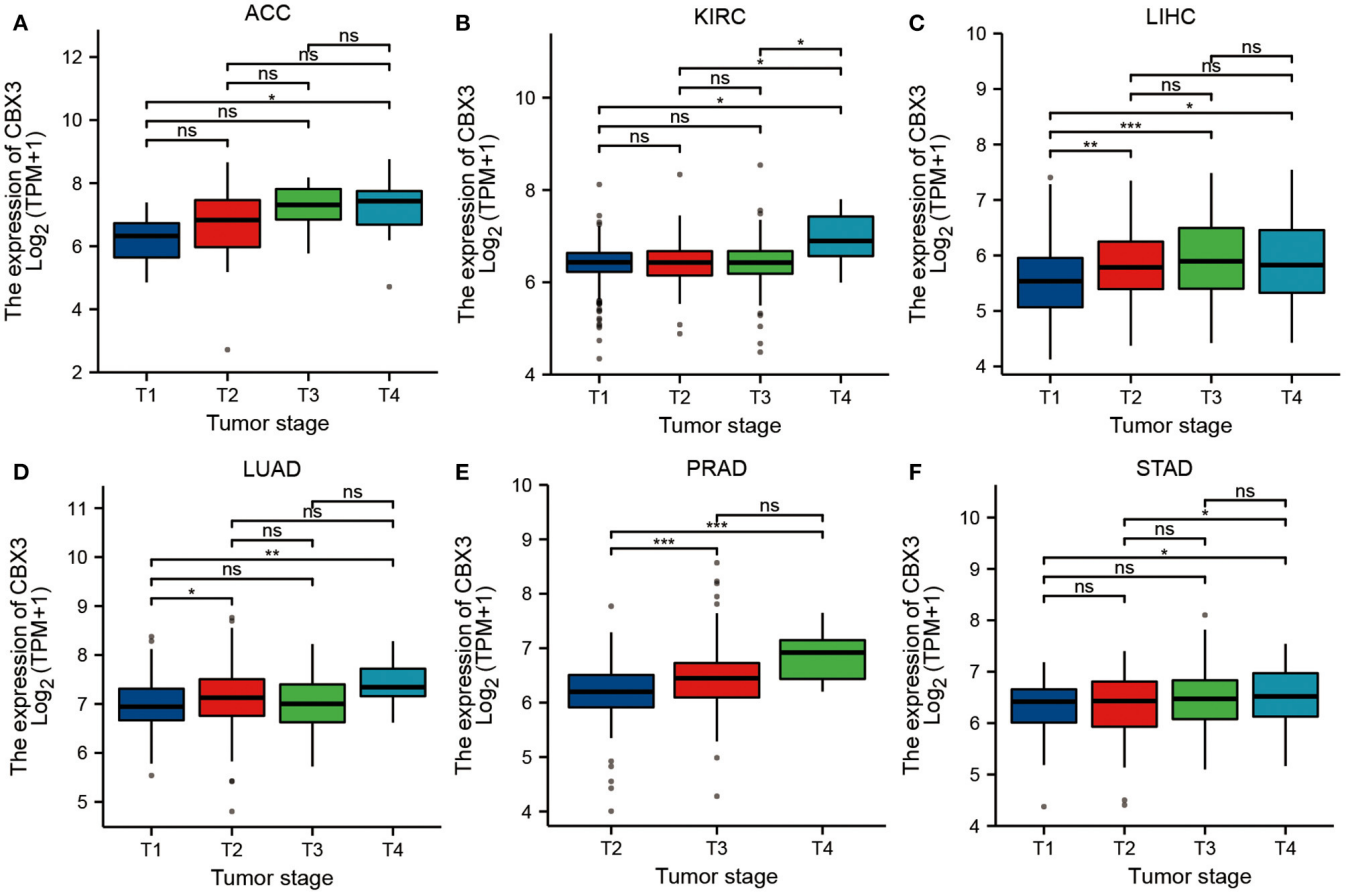
The relationship between the expression of CBX3 and tumor stage in **(A)** ACC, **(B)** KIRC, **(C)** LIHC, **(D)** LUAD, **(E)** PRAD, and **(F)** STAD.

We also assessed the link between the CBX3 expression and tumor stage and discovered that the expression of CBX3 exhibited a considerable correlation with tumor stage in ACC, KIRC, LIHC, LUAD, PRAD, and STAD (Figure 6). However, except for PRAD (Figure 6E), most of the considerable differences in CBX3 expression occurred between stage-one and stage-four tumors, and the expression of CBX3 did not increase along with the increase of tumor stage.

### Correlation of CBX3 Expression Level With TMB and Tumor MSI

**Figure 7:**
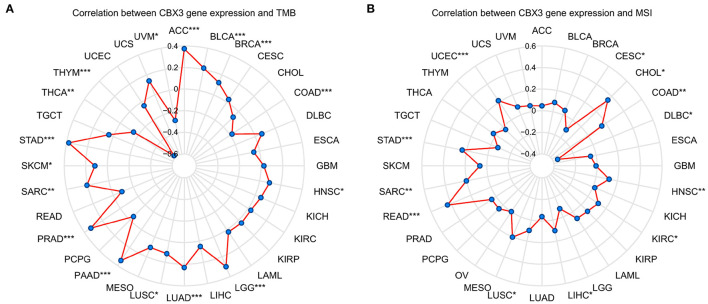
The link between the expression of CBX3 and TMB and MSI. **(A)** Radar diagram demonstrating the correlation between CBX3 and TMB). **(B)** Radar diagram demonstrating the correlation between CBX3 and MSI. *P < 0.05, **P < 0.01, and ***P < 0.001.

Then, we examined whether there is a correlation between the levels of CBX3 expression and MSI and TMB, both of which are considerably associated with the sensitivity of immune checkpoint inhibitors. The outcomes illustrated that CBX3 expression was related to TMB in 16 tumors including ACC, BLCA, BRCA, COAD, and LUAD (Figure 7A). In another 12 tumor types, including nodule COAD, READ, STAD, and UCEC, CBX3 expression correlates with MSI (Figure 7B).

### Relationship Between Expression of CBX3 and Tumor Microenvironment (TME)

**Figure 8:**
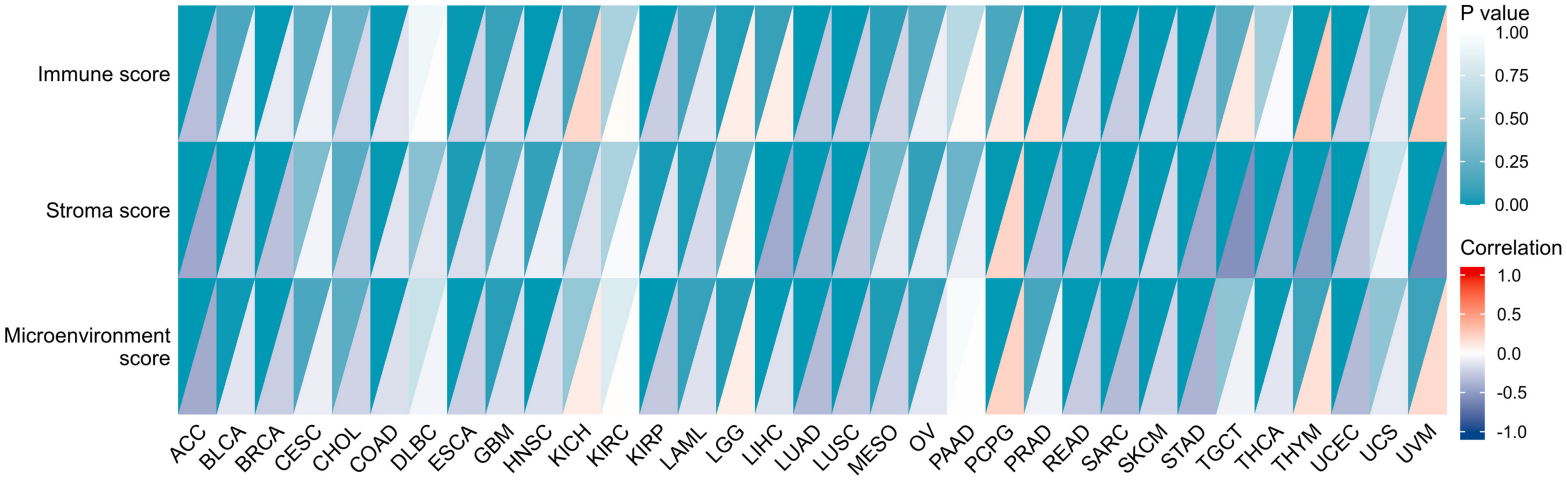
Relationship between the expression of CBX3 and stromal score, immune score, and microenvironment score. Each color block is divided into two triangles, The depth of shade of the lower triangle signifies the magnitude of the correlation coefficient, and the depth of shade of upper triangle represents the the magnitude of *p*-value of the statistical analysis.

More and more reports illustrated that the tumor immune microenvironment has a vital function in tumor genesis and development (21, 22). Thus, it is crucial to additionally examine the link between TME and CBX3 expression. The XCELL algorithm was utilized to compute the immune score, stromal score, as well as, the immune microenvironment score of 33 tumors, and the relationship between the expression level of CBX3 and these three scores were evaluated. The resulting heat map shows (Figure 8) that in addition to serous cystadenocarcinoma of the ovary (OV), acute myeloid leukemia (LAML), CESC, CHOL, testicular carcinoma (TGCT), uterine sarcoma (UCS), KICH, DLBC, KIRC, PAAD, the expression of CBX3 has a negative correlation with the immune score, stroma score and TME score in most tumors.

### Correlation Between the Expression Level of CBX3 and TIICs

**Figure 9:**
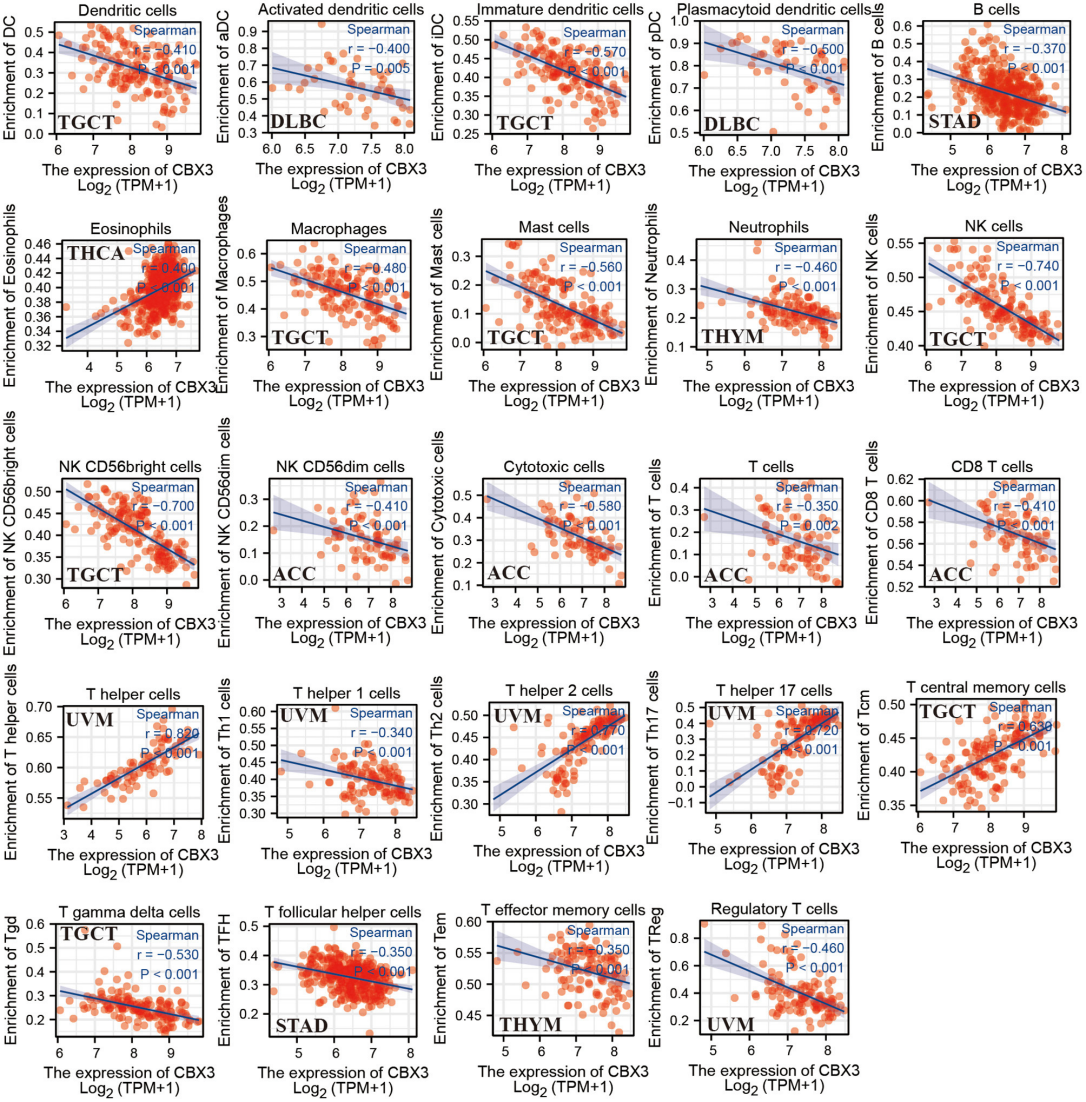
Association between CBX3 expression and different tumor-infiltrating immune cells.

Next, we assessed the link between the level of CBX3 expression and the infiltration level of 24 immune-related cells. The results indicated that the infiltration level of most types of immune cells is significantly associated with CBX3 expression (Supplementary Table 1). Six tumors exhibiting the greatest relationship between the expression of CBX3 and the extent of immune cell infiltration were chosen for additional analysis (Table 1), including BRCA, COAD, LUSC, cutaneous melanoma (SKCM), THYM and UCEC. The expression of CBX3 had a positive correlation with the levels of T helper cells, T memory cells, Tγδ cells and T helper 2 cells in the six tumors analyzed, and a negative correlation with the other 20 immune-related cells. Figure 9 displays the tumors having the greatest relationship between the extent of infiltration of each type of immune cell and the expression of CBX3. The data for other tumors are encompassed in Supplementary Table 1.

**Table 1:** The link between the expression of CBX3 and immune cell infiltration of various types of tumors.

**Cell Type**	**BRCA (*P*-value/Cor)**	**COAD (*P*-value/Cor)**	**LUSC (*P*-value/Cor)**	**SKCM (*P*-value/Cor)**	**THYM (*P*-value/Cor)**	**UCEC (*P*-value/Cor)**
Activated dendritic cells	−0.026	^***^/−0.152	^***^/−0.162	^***^/−0.278	^**^/−0.282	−0.033
B cells	^***^/−0.128	^**^/−0.153	^***^/−0.232	^***^/−0.176	0.038	^***^/−0.149
CD8 T cells	^***^/−0.242	^***^/0.05	^***^/−0.152	^***^/−0.168	^***^/0.392	−0.031
Cytotoxic cells	^***^/−0.229	^**^/−0.208	^***^/−0.156	^***^/−0.357	^***^/−0.468	^***^/−0.277
Dendritic cells	^***^/−0.22	^**^/−0.221	^***^/−0.257	^***^/−0.379	−0.178	^***^/−0.166
Eosinophils	^***^/−0.136	^***^/−0.131	^***^/−0.28	^***^/−0.227	^**^/0.268	^***^/−0.157
Immature dendritic cells	^***^/−0.262	^*^/−0.198	^***^/−0.341	^***^/−0.367	−0.132	^***^/−0.331
Macrophages	^***^/−0.115	^***^/−0.036	^***^/−0.243	^***^/−0.24	^***^/−0.342	^*^/0.087
Mast cells	^***^/−0.187	^**^/−0.116	^***^/−0.316	^***^/−0.149	^***^/−0.317	^***^/−0.175
Neutrophils	^***^/−0.146	^***^/−0.135	^***^/−0.284	^***^/−0.405	^***^/−0.462	^***^/−0.273
NK CD56bright cells	^***^/−0.142	^***^/−0.335	^***^/−0.166	^***^/−0.351	^***^/−0.402	^***^/−0.487
NK CD56dim cells	^***^/−0.138	^***^/−0.092	^***^/−0.206	^***^/−0.392	^***^/−0.325	^***^/−0.207
NK cells	^***^/−0.313	^**^/−0.13	^***^/−0.159	^***^/−0.285	^***^/−0.511	^***^/−0.28
Plasmacytoid dendritic cells	^***^/−0.359	^***^/−0.158	^***^/−0.242	^***^/−0.426	^***^/−0.481	^***^/−0.399
T cells	^***^/−0.138	^*^/−0.177	^***^/−0.173	^**^/−0.273	^***^/0.845	^***^/−0.228
T helper cells	^***^/0.181	0.3	0.08	^***^/0.218	^***^/0.776	^***^/0.274
T central memory	^*^/0.076	^*^/0.253	−0.042	^***^/0.238	^***^/0.478	^***^/0.303
T effector memory	^***^/−0.167	−0.149	^**^/−0.128	^***^/−0.214	^***^/−0.349	^***^/−0.174
T follicular helper	^***^/−0.103	^***^/−0.119	^***^/−0.177	^***^/−0.337	^***^/0.331	^***^/−0.156
T gamma delta	0.622/0.015	^***^/0.092	0.061	^***^/0.242	^*^/−0.206	^**^/0.126
T helper 1 cells	^*^/−0.069	^***^/−0.106	^***^/−0.206	^***^/−0.228	^***^/−0.34	−0.028
T helper 17 cells	^***^/−0.128	^*^/−0.174	^**^/−0.133	^***^/−0.239	^***^/0.72	^***^/−0.218
T helper 2 cells	^***^/0.385	^***^/0.24	^***^/0.203	^***^/0.196	^***^/0.772	^***^/0.487
Regulatory T cells	−0.058	^***^/−0.233	^***^/−0.227	^***^/−0.45	^***^/−0.46	^***^/−0.341

* *P < 0.05*,

** *P < 0.01*,

*** *P < 0.001*.

**Figure 10:**
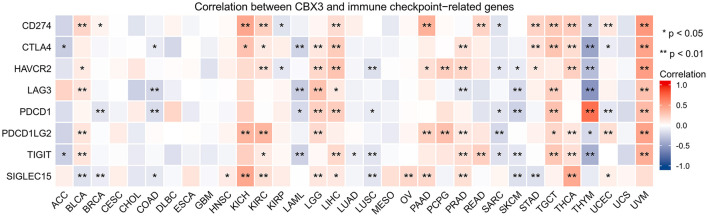
Correlation between the expression of CBX3 and immune checkpoint related genes. *P < 0.05, **P < 0.01.

**Figure 11:**
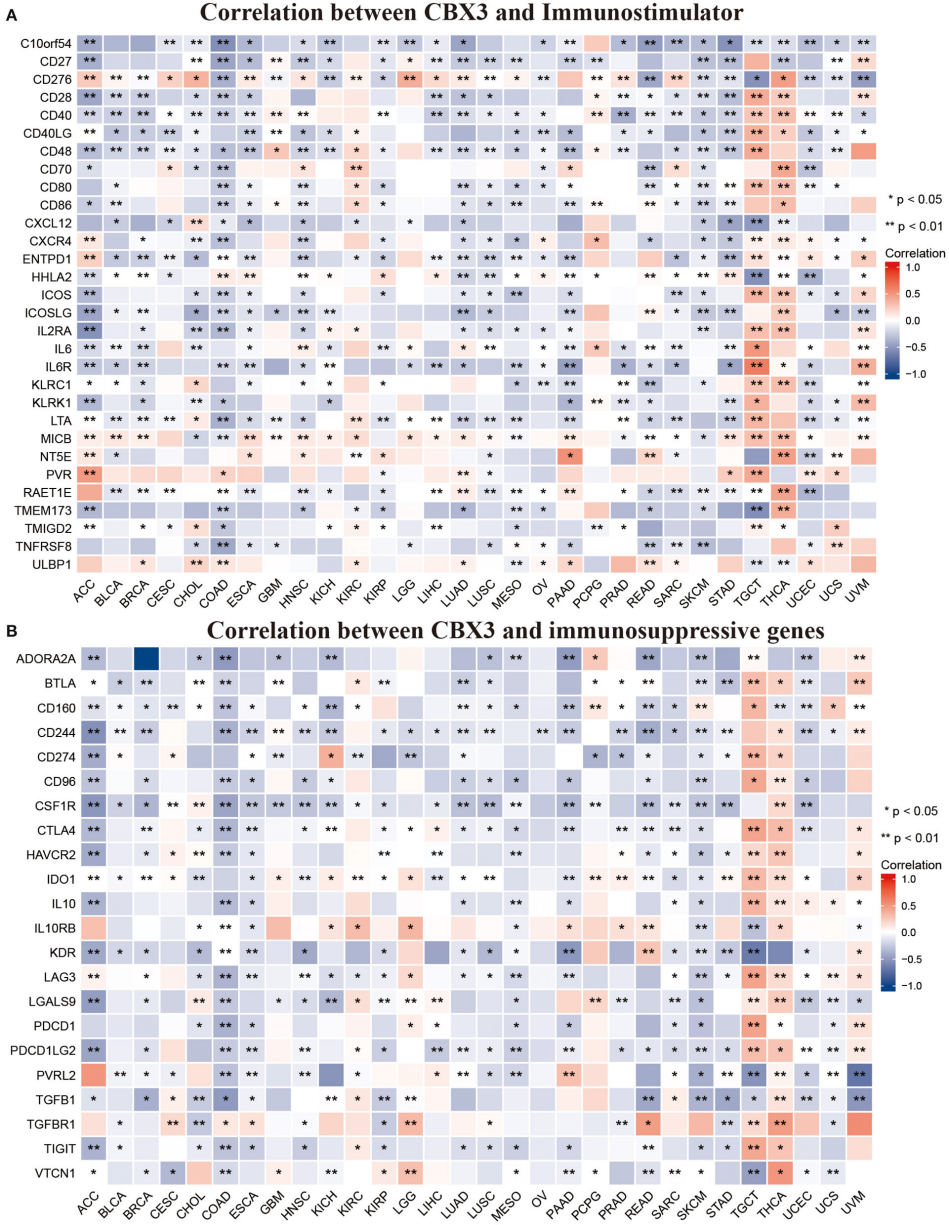
Correlation between CBX3 expression and immunostimulating gene **(A)** and immunosuppressive gene **(B)**. *P < 0.05, **P < 0.01.

In addition, we performed gene correlation analysis to examine the relationship between the expression of CBX3 and immune-associated genes in 33 tumors. The genes evaluated encode immune checkpoint, immune activation, immunosuppression, chemokine, as well as, chemokine receptor proteins. The heat map of immune checkpoint-related genes (Figure 10) showed that nearly all immune checkpoint-associated genes were correlated with CBX3, and most were negatively correlated with CBX3 except BLCA, KICH, KIRC, LGG, LIHC, PAAD, TGCT, THCA, and UVM (*P* < 0.05). Heat maps of immunoactivation, immunosuppression, chemokine and chemokine receptor-related genes showed that most tumor immune-related genes were negatively correlated with CBX3 (*P* < 0.05) except PCPG, TGCT, THCA and UVM (Figure 11, Supplementary Figure 4).

### Correlation Between CBX3 Expression and DNA Methylation

**Figure 12:**
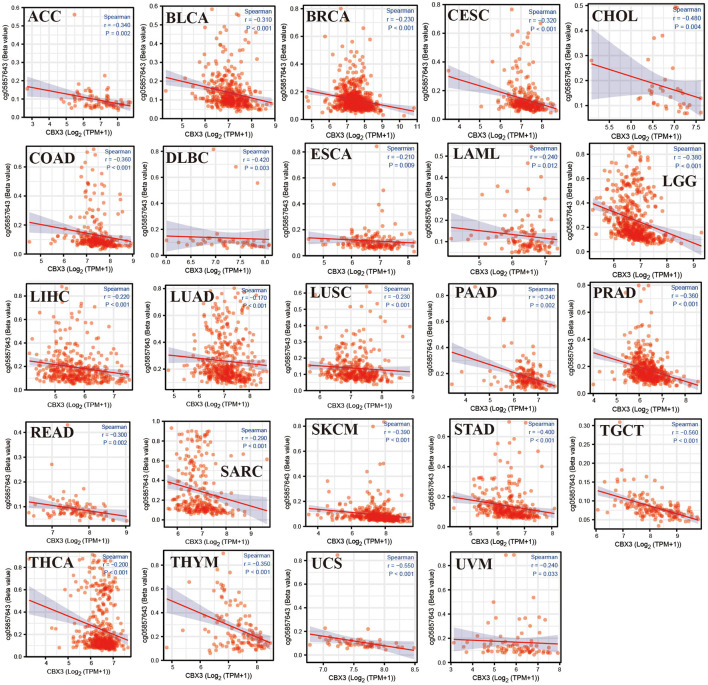
Association between CBX3 expression and promoter methylation of various tumor genes.

**Figure 13:**
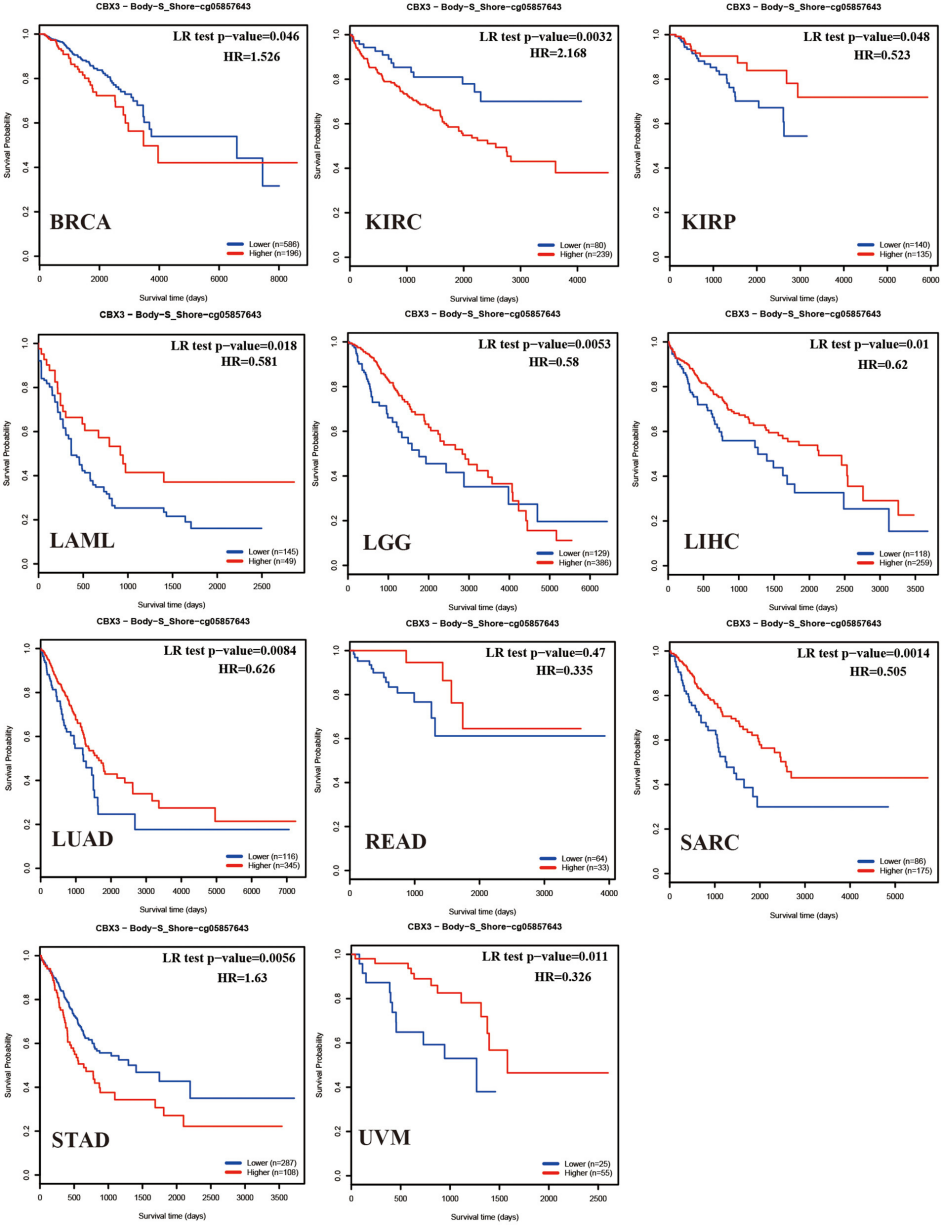
Correlation between CBX3 methylation and OS of various tumors.

The relationship among CBX3 promoter methylation was computed utilizing the cBioPortal database and found a considerable relationship between the expression of the CBX3 gene and methylation in 24 tumors, all of which were negatively correlated (Figure 12). In addition, KM survival analysis was performed to investigate the correlation between CBX3 promoter methylation and the prognosis of patients. In terms of OS, CBX3 methylation is a risk factor for BRCA, KIRC and STAD patients, but a protective factor for KIRP, LAML, UVM, SARC, READ, LUAD, LIHC, and LGG patients (Figure 13).

### GSEA Analysis

**Figure 14:**
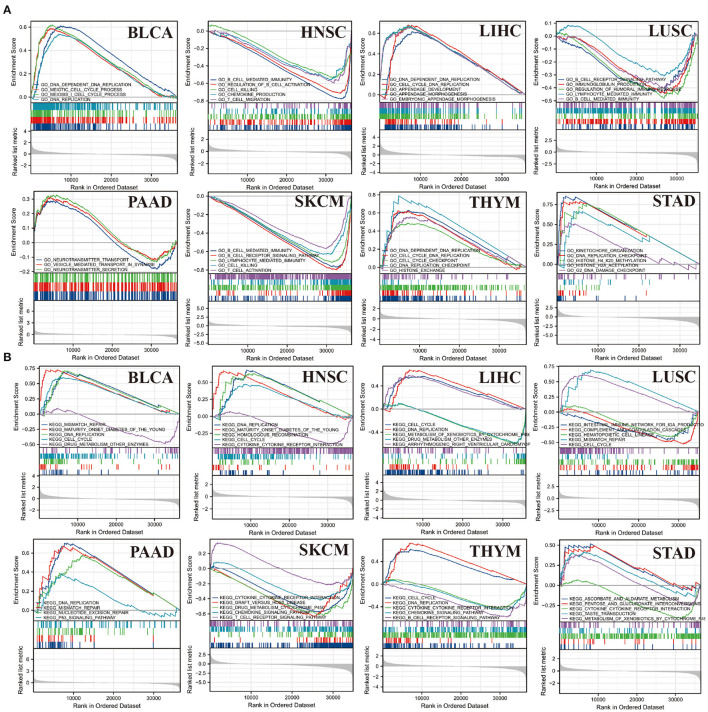
Outcomes of GSEA. **(A)** GO function annotation of CBX3 in a variety of tumors. **(B)** KEGG pathway analysis of CBX3 in a variety of tumors. Dissimilar colored curves display distinct roles or pathways regulated in diverse tumors. Peaks on the downward curve designate negative regulation while Peaks on the upward curve designate positive regulation.

GESA analysis was performed to study the biological value of CBX3 expression in various tumor tissues. The results from the KEGG pathway analysis and GO functional annotation are illustrated in Figure 14. The outcomes indicate that CBX3 positively modulates DNA replication checkpoints and cell cycle checkpoints and other metabolic processes in ACC, LIHC, THYM, and STAD. In contrast, CBX3 negatively regulates a variety of immune-related functions, such as immune response, B/T cell activation, immune regulation and signaling pathways, in HNSC, LUSC and SKCM (Figure 14A). The expression of CBX3 in BLCA, LIHC, LUSC, HNSC and STAD was positively correlated with cell cycle and drug metabolism pathway. However, in LUSC, SKCM, THYM, and STAD, CBX3 expression was negatively correlated with cytokine and chemokine signaling pathways, as well as several immune-related pathways, including graft-versus-host disease, natural killer cell-mediated cytotoxicity and B/T cell receptor signaling pathways (Figure 14B).

## Discussion

As medical technology continues to advance, clinicians could increase the curative efficacy of cancer patients through surgery, targeted therapy, endocrine therapy, or chemotherapy (23, 24). However, tumor management is not always adequate. Tumor targets or characteristics must be identified for personalized treatment to elevate the chances of curing cancer patients (25).

Past research reports have assessed the expression level and role of CBX3 in many tumors, including glioblastoma (26) and gastric cance (27). However, the role of CBX3 in different tumors and whether it might function as a prognostic marker is yet to be investigated. In this research, we comprehensively examined the expression of CBX3 in various tumors. The results of this research illustrated that CBX3 was highly expressed in 29 tumors, and was considerably correlated with DNA methylation, MSI, and TMB. Additionally, the overexpression of CBX3 is linked to dismal prognosis (PFI, DSS, and OS) in a variety of tumors. Moreover, the CBX3 expression is strongly linked to the level of immune invasion and immune-related genes in human pan-cancer, especially in ACC, MESO, LUAD, LIHC, LGG, HNSC, and PAAD. The outcomes also indicate that CBX3 has a critical function in tumor immunity and might serve as an imperative biomarker. These findings are consistent with results from other recently reported studies (28).

Past research reports have also indicated that CBX3 is a crucial component in the development of certain types of tumors. Zhang et al. showed that CBX3 was up-regulated in tongue squamous cell carcinoma and postponed the G1/S phase through down-regulation of P21, which promoted tumor proliferation and had adverse effects on prognosis (29). Chen et al. found that CBX3 promotes cell cycle conversion by inhibiting FBP1 in pancreatic cancer, which leads to tumor progression (11). Recent evidence from studies has shown that CBX3 is upregulated in colorectal cancer in human, which stimulated cell proliferation by candidly modulating CDKN1A through methylation of histone H3K9 at its promoter (30). In osteosarcoma (31), the CBX3 overexpression could regulate cell proliferation by stimulating the transformation of the G1/S cell cycle. Moreover, CBX3 Knockdown was found to impedes the proliferation of cancer cells in glioblastoma by stagnating the cell cycle during G2/M cell cycle transition (26). Alam et al. discovered that CBX3 promoted proliferation, formation of colony and migration of LUAD cells via the direct inhibition of NCOR2 and ZBTB7A (32). These studies indicate that CBX3 has an extremely imperative function in the development of tumors.

Furthermore, we discovered that the expression of CBX3 expression is related to age in certain tumor types. CBX3 has a lower expression level in youthful patients with LUSC, THCA, LUAD, ESCA, HNSC, KIRP, and THYM, while decreased CBX3 expression is linked to elderly STDA patients. These outcomes might have great implications in guiding patients of different age groups as they select their appropriate immunotherapy. This research also illustrated that the expression of CBX3 was linked to several tumor stages, especially between stage-I and stage-IV tumors. Past research reports have indicated that CBX3 expression has a positive correlation with tumor stage in patients with stomach adenocarcinoma (33).

DNA methylation, which has been recognized as the main kind of DNA epigenetic modification, is involved in the regulation of gene expression devoid of any alteration in the DNA sequence (33). Usually, DNA methylation inhibits gene expression through the alteration of the DNA conformation, DNA stability, and chromatin structure (34). In recent decades, the connection between DNA methylation and tumors has been gradually uncovered. Hypermethylation within the promoter region often results in the inactivation or silencing of tumor suppressor genes in tumor cells (34, 35). This research illustrated that DNA promoter methylation was correlated with CBX3 expression in most common tumors. In terms of OS, CBX3 methylation level is a risk factor for BRCA, KIRC and STAD patients.

TMB is an auspicious marker to predict pan-cancer, which might offer guidance to immunotherapy particularly in the current era of targeted therapies (36). Past studies have illustrated that TMB might be utilized as a biomarker to enhance immunotherapy for colorectal cancer (37) and non-small cell lung cancer (38). In addition, TMB might forecast the prognosis of patients with generalized cancer following immunotherapy (39). MSI has also been recognized as an essential biomarker of immune checkpoint inhibitors (37). The elevated frequency MSI observed in colorectal cancer is not only of diagnostic value but also an independent prognostic indicator (40). Our research illustrated that CBX3 expression was linked to TMB in 16 kinds of tumors and MSI in 12 kinds of tumors. This could suggest that the levels of TMER2 expression impact MSI and TMB of tumor, and thus affect the reaction to immune checkpoint inhibitor therapy of patients. Our research offers a novel foundation for the prognosis of immunotherapy. Premised on current studies and the findings from our research, we deduce that among the tumors whose CBX3 expression is positively correlated with TMB, elevated MSI and TMB might provide an improved prognosis following immune checkpoint inhibitor treatment.

At present, TME has always been the main focal point and trend in tumor research. Notably, immune cells are an imperative component of TME. The function of immune cells is distinct owing to the expression of a number of key modulators, such as microRNAs (41). In general, immune cells show strong anti-tumor characteristics. Cumulative research reports have illustrated that immune cells in TME might have a crucial function in the development and progression of a variety of tumors (42, 43). Nevertheless, few studies have revealed the function of CBX3 in TME. According to the outcomes of this research, the CBX3 expression is increasingly related to invasive immune cells in BRCA, COAD, LUSC, SKCM, THYM, and UCEC tumors. In addition, according to the xCELL score, CBX3 expression had a negative correlation with the stromal score, immune score and microenvironment score of most tumors. In addition, we found that CBX3 was linked to the expression of eight immune checkpoint indicators in different tumors, especially LIHC, THYM, and UVM. Also, our study revealed that CBX3 is correlated with genes of immune activation, immunosuppression, chemokine and chemokine receptor proteins. These outcomes illustrate that the CBX3 expression is strongly correlated with the immune invasion of tumor cells, which impacts the prognosis of patients and provides a novel target for the exploitation of immunosuppressors.

Moreover, the enrichment analysis indicates that CBX3 may affect tumor etiology or pathogenesis through the following ways: cell cycle, signaling pathways, immune regulation, immune response, and B/T cell activation. These results are harmonious with those of past research reports. Sun et al. (44) found that CBX3-deficient mice treated with CD8 + T cells can induce changes in tumor immune environment, reduced tumor burden, and subsequently inhibited tumor growth.

In conclusion, this research provided the pan-cancer analysis of CBX3 and illustrated the differential expression of CBX3 between tumors and non-tumor normal tissues. The research also uncovers the correlation among CBX3 expression, DNA methylation and clinical prognosis. The results indicate that CBX3 could be utilized as an independent prognostic indicator for numerous tumors. Premised on diverse tumors, the CBX3 expression levels generates distinct prognostic outcomes, and the precise function of CBX3 in each of these tumor needs to be further studied. In addition, CBX3 expression is linked to immune cell infiltration in diverse kinds of tumors, MSI, and TMB. Its influence on tumor immunity is varied premised on the type of tumor. These results could help illuminate the function of CBX3 in tumor genesis as well as development, and offer a foundation for more accurate and individualized immunotherapy in future.

## Data Availability Statement

The datasets presented in this study can be found in online repositories. The names of the repository/repositories and accession number(s) can be found in the article/Supplementary Material.

## Ethics Statement

We performed secondary analyses on the data from The Cancer Genome Atlas (TCGA) that is publically available at an open resource web platform, the Genomic Data Commons Data Portal (https://portal.gdc.cancer.gov/).

## Author Contributions

All authors were involved in data collection, drafting, graphics, and final manuscript. CJ revised the whole manuscript, worked the new figures, and critically improved the text. HX supervised, provided resources, and wrote the final draft. All authors contributed to the article and approved the submitted version.

## Funding

HX is supported by the School Research Funding of Anhui Medical University (2021xkj237 and 2019xkj003).

## Conflict of Interest

The authors declare that the research was conducted in the absence of any commercial or financial relationships that could be construed as a potential conflict of interest.

## Publisher's Note

All claims expressed in this article are solely those of the authors and do not necessarily represent those of their affiliated organizations, or those of the publisher, the editors and the reviewers. Any product that may be evaluated in this article, or claim that may be made by its manufacturer, is not guaranteed or endorsed by the publisher.

## Supplementary Material

Supplementary Figure 1 Contrast of CBX3 expression between tumor and paired non-tumor normal samples (AUC < 0.9).

Supplementary Figure 2 The link between the level of CBX3 expression and DSS. **(A)** Forest map of the link between CBX3 expression and DSS in 33 kinds of tumors. **(B–J)** KM analysis of the correlation between CBX3 expression and DSS.

Supplementary Figure 3 The link between CBX3 expression and PFI. **(A)** Forest map of PFI and CBX3 expression in 33 kinds of tumors. **(B–H)** KM analysis of the link between CBX3 expression and PFI.

Supplementary Figure 4 Association between CBX3 expression and chemokine **(A)** and chemokine receptor **(B)** genes. ^*^*P* < 0.05, ^**^*P* < 0.01.

Supplementary Table 1 The association between the expression of CBX3 and different immune cell infiltration in the other 27 tumors.
