# Supplementary figures and images for: Analysis of Pan-Cancer Revealed the Immunological and Prognostic Potential of CBX3 in Human Tumors
Source: Front Med (Lausanne). 2022 Apr 28;9:869994. doi: 10.3389/fmed.2022.869994 (PMC9096250; doi:10.3389/fmed.2022.869994)

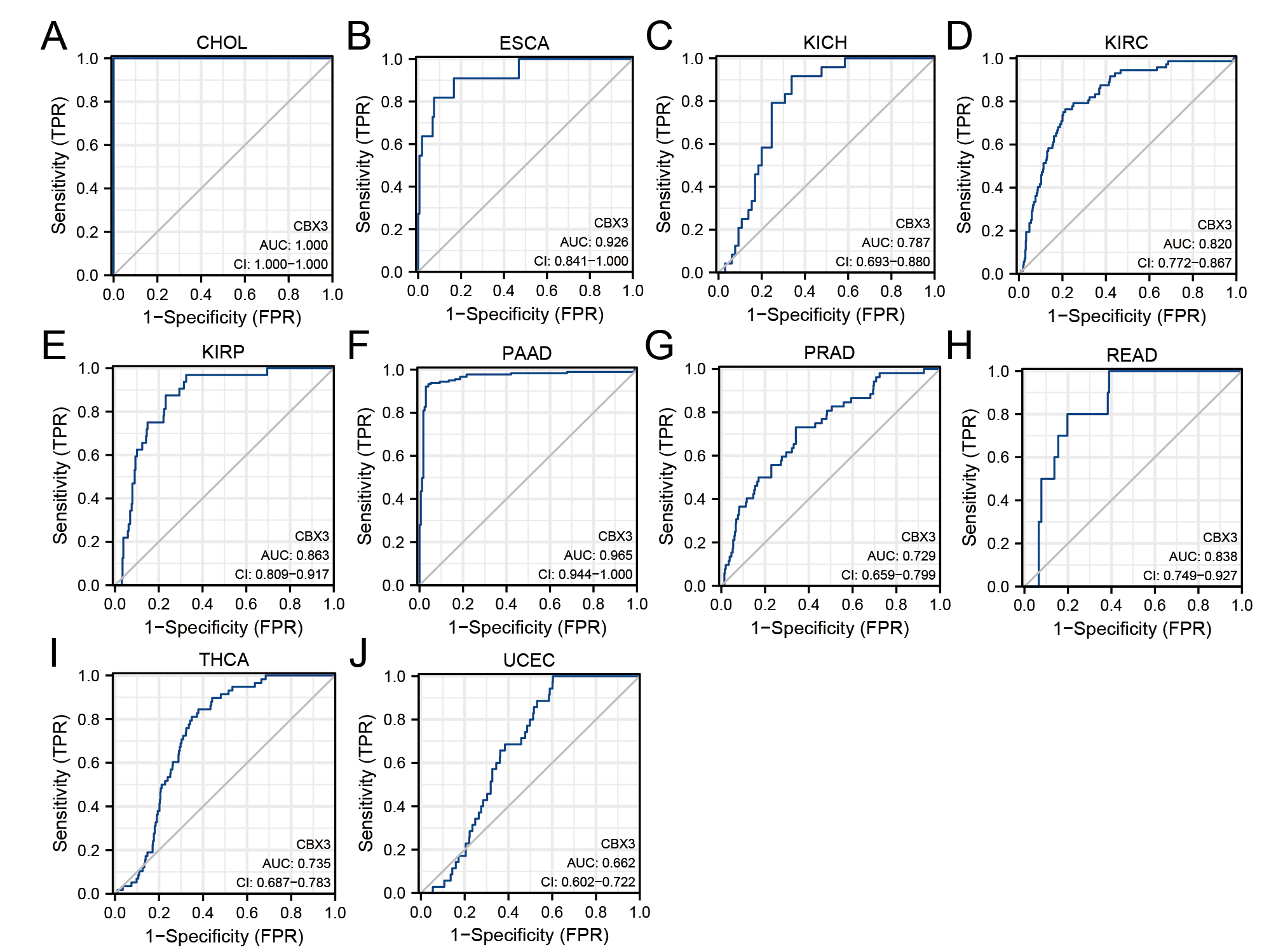

Supplement: Supplementary Figure 1 — Contrast of CBX3 expression between tumor and paired non-tumor normal samples (AUC < 0.9). [file Image_1.TIF]

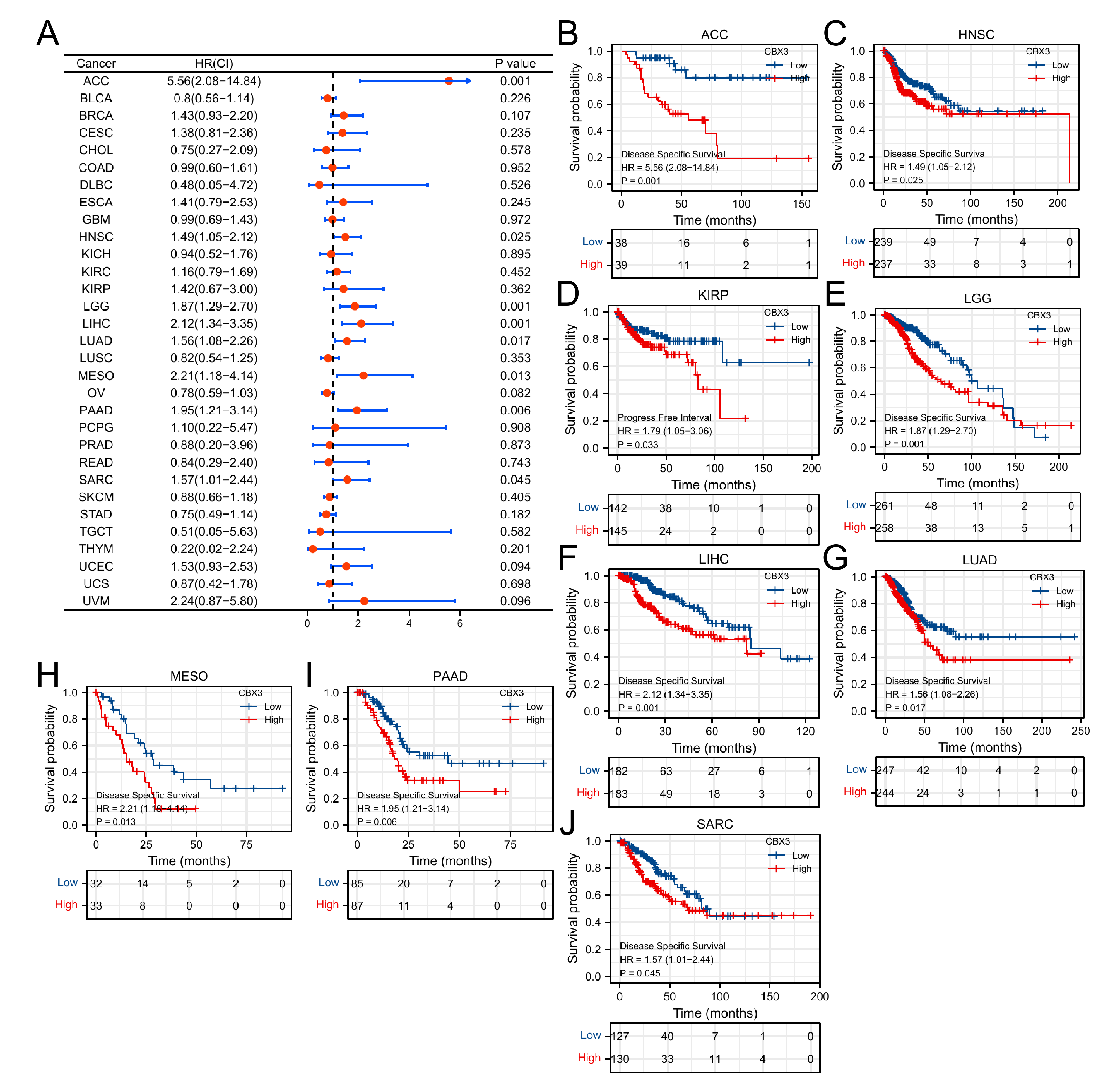

Supplement: Supplementary Figure 2 — The link between the level of CBX3 expression and DSS. (A) Forest map of the link between CBX3 expression and DSS in 33 kinds of tumors. (B–J) KM analysis of the correlation between CBX3 expression and DSS. [file Image_2.TIFF]

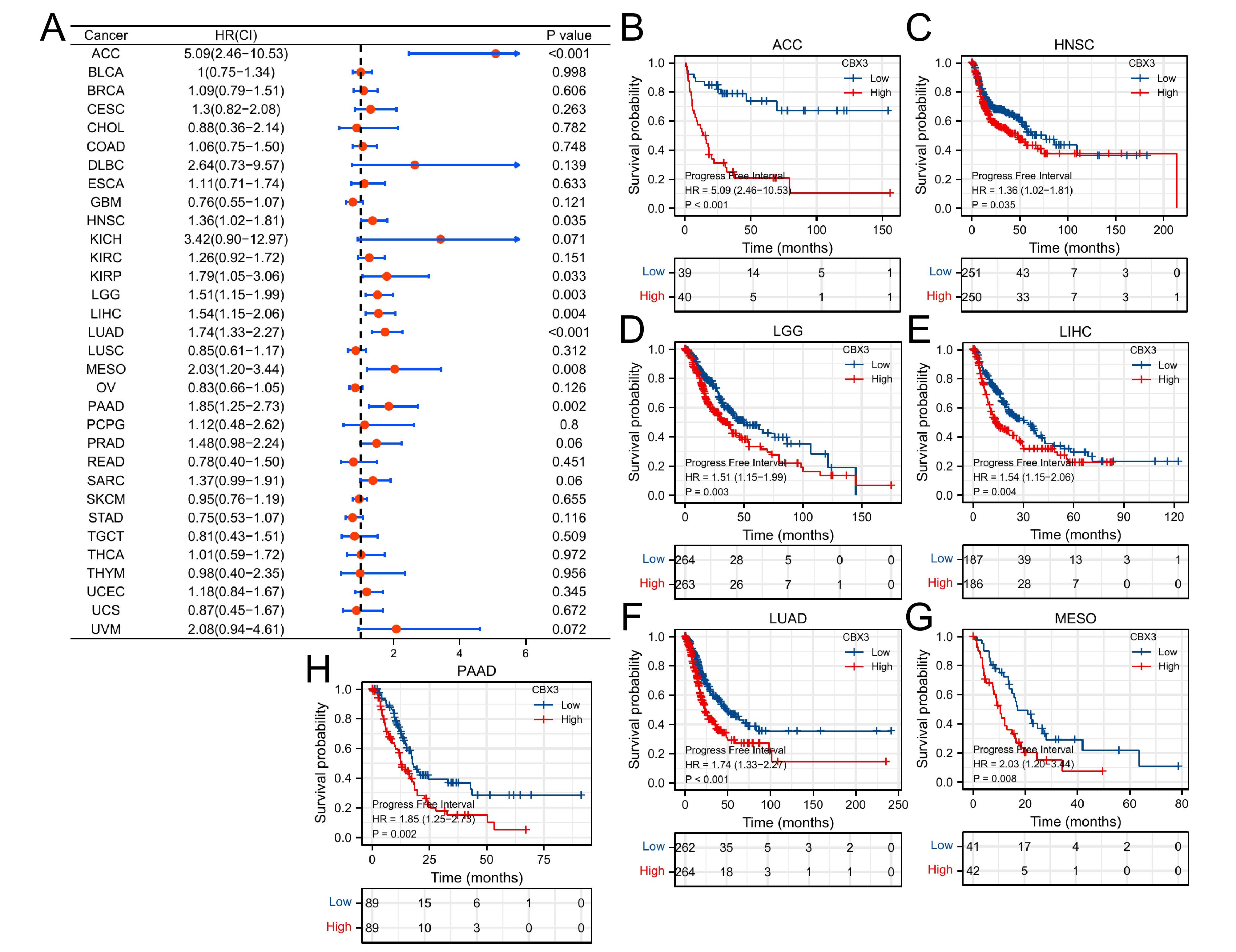

Supplement: Supplementary Figure 3 — The link between CBX3 expression and PFI. (A) Forest map of PFI and CBX3 expression in 33 kinds of tumors. (B–H) KM analysis of the link between CBX3 expression and PFI. [file Image_3.TIFF]

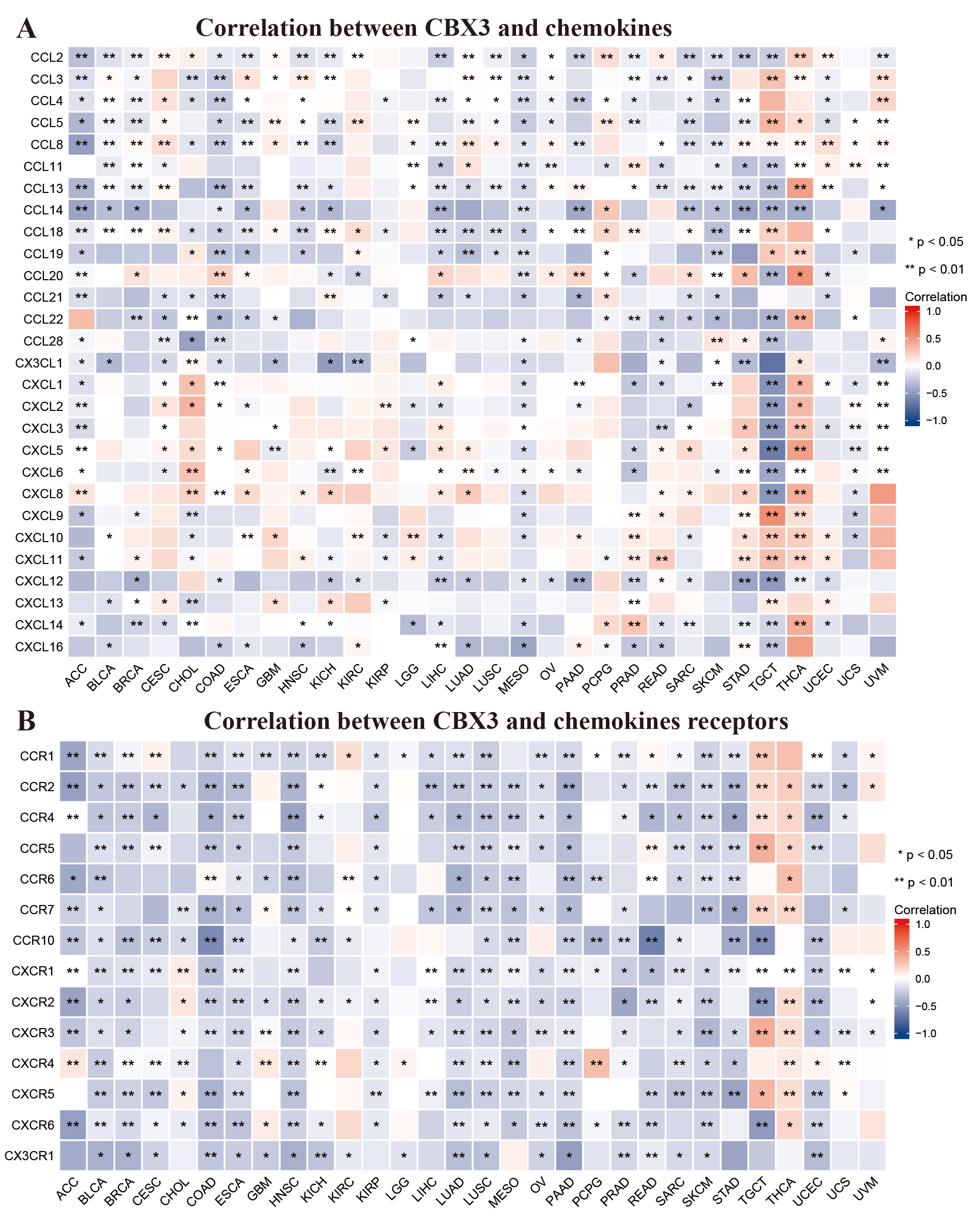

Supplement: Supplementary Figure 4 — Association between CBX3 expression and chemokine (A) and chemokine receptor (B) genes. *P < 0.05, **P < 0.01. [file Image_4.TIF]
